# Light Controls Growth and Development via a Conserved Pathway in the Fungal Kingdom

**DOI:** 10.1371/journal.pbio.0030095

**Published:** 2005-03-15

**Authors:** Alexander Idnurm, Joseph Heitman

**Affiliations:** **1**Department of Molecular Genetics and Microbiology, Howard Hughes Medical InstituteDuke University Medical Center, Durham, North CarolinaUnited States of America; The Salk Institute for Biological StudiesUnited States of America

## Abstract

Light inhibits mating and haploid fruiting of the human fungal pathogen *Cryptococcus neoformans,* but the mechanisms involved were unknown. Two genes controlling light responses were discovered through candidate gene and insertional mutagenesis approaches. Deletion of candidate genes encoding a predicted opsin or phytochrome had no effect on mating, while strains mutated in the *white collar 1* homolog gene *BWC1* mated equally well in the light or the dark. The predicted Bwc1 protein shares identity with Neurospora crassa WC-1, but lacks the zinc finger DNA binding domain. *BWC1* regulates cell fusion and repression of hyphal development after fusion in response to blue light. In addition, *bwc1* mutant strains are hypersensitive to ultraviolet light. To identify other components required for responses to light, a novel self-fertile haploid strain was created and subjected to *Agrobacterium*-mediated insertional mutagenesis. One UV-sensitive mutant that filaments equally well in the light and the dark was identified and found to have an insertion in the *BWC2* gene, whose product is structurally similar to N. crassa WC-2. The C. neoformans Bwc1 and Bwc2 proteins interact in the yeast two-hybrid assay. Deletion of *BWC1* or *BWC2* reduces the virulence of C. neoformans in a murine model of infection; the Bwc1-Bwc2 system thus represents a novel protein complex that influences both development and virulence in a pathogenic fungus. These results demonstrate that a role for blue/UV light in controlling development is an ancient process that predates the divergence of the fungi into the ascomycete and basidiomycete phyla.

## Abstract 2

Two genes controlling light responses - *BWC1* and *BWC2* - were identified and shown to regulate development and virulence of the fungal pathogen *Cryptococcus neoformans*

## Introduction

Light is the fundamental energy source for life on earth and as such is a major environmental signal for organisms from all kingdoms of life. In the fungal kingdom, light can regulate growth, the direction of growth, asexual and sexual reproduction, and pigment formation, all of which are important aspects for the survival and dissemination of fungal species. These processes have negative implications to many aspects of human life, as the uncontrolled proliferation of fungi can lead to devastating plant disease, mold, and human disease. On the other hand, fungi are essential for recycling nutrients in the environment, in mycorrhizal interactions with plants, and as a source of food and pharmaceutical metabolites for humans. Understanding the role of environmental signals in fungal development is vital to increase the benefits and decrease the costs that fungi present. Despite the importance of light to fungal development, much has yet to be determined to illuminate the mechanisms fungi use to perceive and respond to light.

The effects of light have been investigated in model fungal species. While spectral analyses and the morphological effects of light have been well characterized in genera such as *Coprinus* (a basidiomycete) or *Phycomyces* (a zygomycete), at the molecular level Neurospora crassa (an ascomycete) is best understood based on the functions of the *white collar* (*wc-1* and *wc-2*) genes in light sensing [1,2,3]. In *N. crassa,* blue light regulates induction of carotenoid pigment production, protoperithecia (sexual fruiting body) formation and phototropism of perithecial beaks, and circadian rhythm, all of which are abolished by mutations in *wc-1* or *wc-2* [4]. These two genes encode proteins with several conserved domains, including a zinc finger DNA binding domain found in both proteins [5,6,7]. The two proteins physically interact through PAS (conserved in Per, Arnt, Sim proteins) domains [8,9,10]. The WC-1 protein functions as the blue light receptor through a specialized PAS domain responsible for sensing light, oxygen, and voltage in other proteins (LOV domain), and, together with WC-2, acts as a transcription factor. The WC-1 protein interacts with a flavin chromophore [flavin adenine dinucleotide (FAD)] to function as the blue light receptor [11,12]. A small protein, VIVID, also perceives blue light through a LOV domain and modulates N. crassa sensitivity to light [13]. N. crassa has an additional four candidate photoactive protein homologs whose functions in photoperception remain elusive [14,15].

We set out to identify genes involved in the process by which light inhibits mating of the basidiomycete Cryptococcus neoformans. In nature, cryptococcal varieties are associated with bird excreta, soil, tree hollows, and even caves [16,17]. Thus, the light stimuli studied under laboratory conditions are highly relevant to the varying light signals the fungus experiences in the wild. C. neoformans exists as a haploid yeast with two bipolar mating types (**a** and α). MAT**a** and MATα cells fuse to form a dikaryotic hypha that terminates in a basidium in which nuclear fusion and meiosis occur, producing four long chains of haploid basidiospores by mitosis and budding. A similar process, known as haploid or monokaryotic fruiting, can occur with only one mating partner that also gives rise to filaments that terminate in basidium-like structures and produce short spore chains. Spores have been implicated as an infectious propagule, further underscoring the importance of understanding the regulatory processes governing basidiospore production [18,19]. Mating and fruiting are controlled in the laboratory by stimuli such as the presence of potential mating partners (via pheromone signaling), nutrient limitation, desiccation, temperature, and light [19]. Many aspects of the transduction pathways for these signals have been elucidated, particularly in response to pheromones and nutrient limitation [20], but no components of light signaling had been reported to date for this important human pathogen. We identify here two genes required for C. neoformans responses to light, and demonstrate their role in blue light regulation of development and sensitivity to UV light, and their requirement for full virulence of this pathogen in a mammalian host.

## Results

### C. neoformans Expresses Three Candidate Photoreceptors That Could Regulate Development

**Figure 1:**
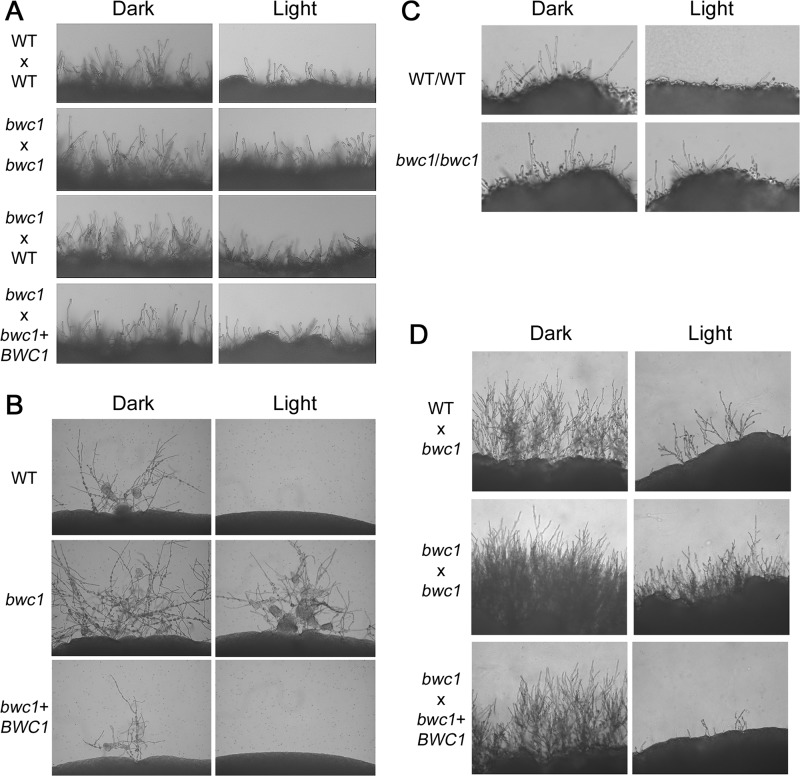
Bwc1 Inhibits Filament Formation during C. neoformans Mating or Haploid Fruiting Filamentation assays were on V8 medium (pH 7) and conducted in the dark or under white fluorescent light. (A) Filamentation in crosses between wild type (WT), *bwc1* mutant, and *bwc1* + *BWC1* reconstituted strains (48 h). Filament formation develops and is overgrown by yeast cells in wild-type or reconstituted strains crossed in the light. (B) Haploid fruiting filaments and blastospore production from yeast colonies incubated on filament agar (7 d). (C) Filament formation in wild-type (WT) or *bwc1*/*bwc1* mutant diploid strains (24 h). (D) Filamentation in crosses of serotype A strains wild type (WT), *bwc1* mutant, and *bwc1* + *BWC1* reconstituted strains mated with a serotype D *bwc1* mutant (48 h).

Mating and fruiting of C. neoformans can be variable during culturing. Previous work in our laboratory and others has endeavored to find environmental factors that lead to this variation. One factor identified was light; cultures wrapped in aluminum foil exhibited enhanced mating and haploid fruiting compared to cultures in the light [19,21]. Our assays used cardboard containers in which 9-cm^2^ holes were excised from the lid and overlaid with aluminum foil or clear plastic wrap. Under these conditions, light inhibited both mating and haploid fruiting of *C. neoformans,* thereby ruling out any effects of plate-sealing on CO_2_ levels or desiccation (Figure 1A and 1B).

In a candidate gene approach, the C. neoformans genome was searched for homologs of fungal genes implicated in light signaling or transduction: *wc-1* and *wc-2, opsin, phytochrome, cryptochrome, vivid, frequency,* and *photolyase*. Unambiguous matches were obtained to *opsin* (*OPS1;* GenBank AY882440), *phytochrome* (*PHY1;* GenBank AY882439), and *white collar 1* (*BWC1;* GenBank AY882437), and transcription of these genes was confirmed by RT-PCR. Opsins are a class of seven-transmembrane proteins that bind retinal via a conserved lysine residue to form ion pumps or light receptors in animals and Archaea. Opsins have been identified in the genomes of a number of fungi, but as yet have no known function [15,22,23,24]. Phytochromes are histidine or serine/threonine kinase red/far-red light receptors identified in plants, and more recently in bacteria (reviewed in [25]). Two phytochrome homologs have been noted in the genome of *N. crassa,* but also have as yet no known function [14]. The predicted *wc-1* homolog contains a LOV domain, two additional PAS domains, and a nuclear localization signal. In contrast to the *N. crassa wc-1* gene, the C. neoformans homolog has no DNA binding domain; our designation of the gene as *Basidiomycete White Collar 1 (BWC1)* is meant to reflect this unique structure.

### *BWC1* Mediates Inhibition of Mating by Light

The three putative photoreceptor genes were mutated in a C. neoformans var. *neoformans* (serotype D) strain by replacing the coding region with the *URA5* gene. Single-, double-, and triple-mutant strains were isolated following genetic crosses to test for possible redundant functions in light sensing. The abilities to mate and to haploid-fruit in the light and dark were examined by monitoring the production of filaments. There was no effect on mating or fruiting in strains with the opsin *ops1* or phytochrome *phy1* mutations, either alone or in combination (unpublished data). In contrast, *bwc1* mutants were insensitive to light. All crosses in which both mating partners (bilateral crosses) carried the *bwc1* deletion showed equivalent mating in the light and the dark, as assessed by the production of filaments after 24 h and 48 h, while in strains with a wild-type copy of *BWC1,* light reduced mating (Figure 1A). In unilateral crosses, i.e., in which a *bwc1* mutant strain was crossed to wild type, only a very modest increase in filamentation in the light was observed relative to crosses between two wild-type parents. The *bwc1* mutant strains exhibited more haploid fruiting in the presence of light compared to *BWC1* wild-type strains, and a somewhat higher level of fruiting in the dark (Figure 1B). Reintroduction of a wild-type copy of *BWC1* into a *bwc1* mutant strain restored the inhibition of mating and fruiting by light (Figure 1). Thus, of the three candidate photoreceptor genes identified, *BWC1* functions in the control of mating and fruiting by light, whereas *OPS1* and *PHY1* do not.

### *BWC1* Controls Cell Fusion and Filament Development

**Figure 2:**
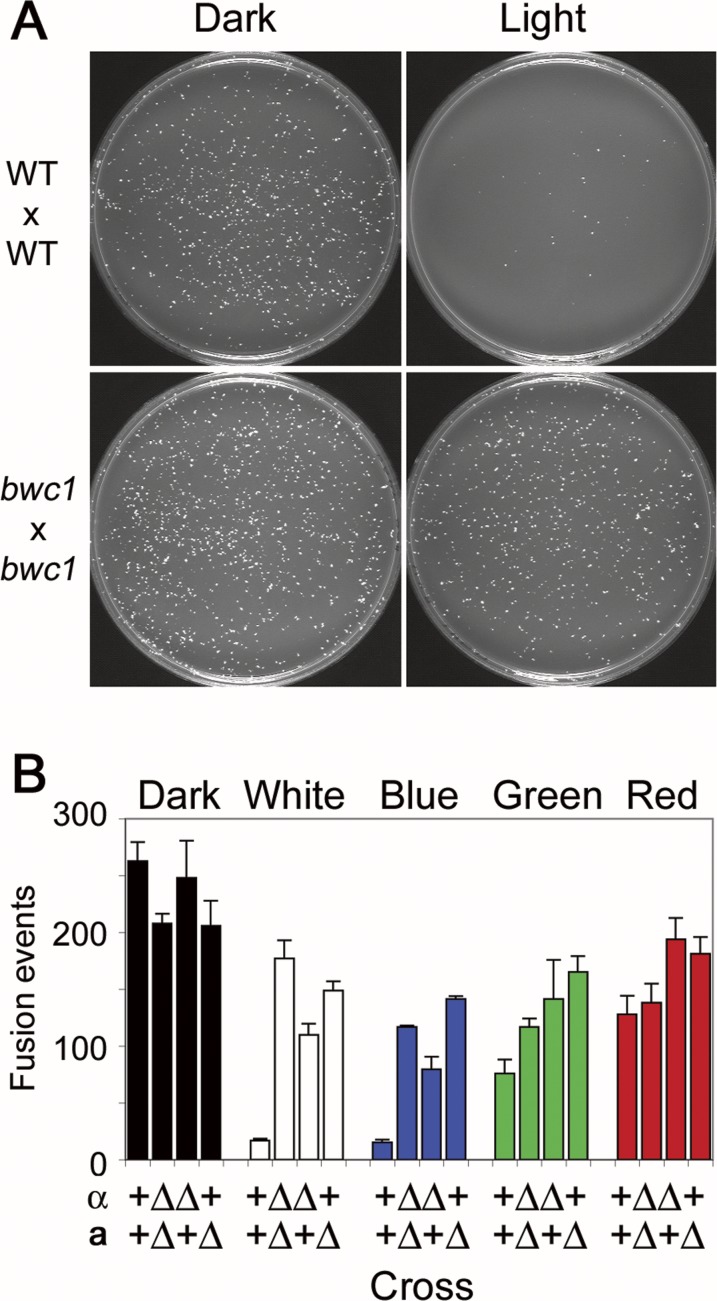
Bwc1 Regulates Fusion of C. neoformans Cells in Response to Blue Light (A) Auxotrophic strains that were wild type (WT) or *bwc1* mutant were mated on V8 medium for 24 h and plated onto minimal medium to select for dikaryotic strains that result from cell fusion events. Light inhibits fusion in wild-type strains, and this inhibition is absent in *bwc1* mutant strains. (B) Fusion efficiency of strains under different wavelengths of light. Fusion is reduced by white or blue light. Matings were between wild-type (+) parents, *bwc1* mutant (Δ) parents, or one wild-type and one mutant parent (*bwc1* α × WT **a**, or WT α × *bwc1* **a**). Mutation of *bwc1* in either or both mating partners relieves inhibition of fusion by white or blue light. Bars indicate the standard error of the mean of three replicates.

Because filament formation is a qualitative rather than a quantitive phenotype, auxotrophic derivatives of *BWC1* wild-type and *bwc1* mutant strains were created, and cell fusion was assayed quantitatively; fusion of two auxotrophic parents (*ade2* or *lys1*) yields prototrophic dikaryotic or diploid strains that can grow on medium lacking adenine and lysine. By this assay, fusion of *bwc1* mutant or wild-type strains was equivalent in the dark. However, light reduced fusion of wild-type strains 10- to 15-fold but had no impact on fusion of the *bwc1* × *bwc1* or *bwc1* × wild-type crosses (Figure 2, and unpublished data).

Light also inhibited self-filamentous growth of a MAT**a**/MATα diploid strain. Stable diploid strains were selected after cell fusion by incubation of strains at 37 °C. Filamentation in the light and dark was examined using diploid wild-type or *bwc1* mutant heterozygous or homozygous strains. The *bwc1*/*bwc1* mutant strain filamented equally well in the light and the dark, whereas filament development was reduced in the wild-type diploid strain in the light (see Figure 1C). The filamentation of strains heterozygous at the *BWC1* locus was inhibited by light, indicating that the *bwc1* mutation is recessive (unpublished data). These results demonstrate that *BWC1* functions in light responses at both the initial cell fusion step and during subsequent filament formation.

### Blue Light Inhibition of Cell Fusion Requires *BWC1*

To determine the approximate wavelengths of light that inhibit mating of *C. neoformans,* cell fusion was assayed during growth under colored filters at 24 and 48 h. In four independent experiments, blue light was sufficient to inhibit cell fusion, whereas green or red wavelengths had little or no effect (see Figure 2B for a representative experiment). In all crosses, only one mating parent required a *bwc1* mutant allele to escape the inhibition of cell fusion by light. Thus, C. neoformans Bwc1 functions similarly to the N. crassa WC-1 protein in response to blue light.

### *BWC1* Is Required for UV Resistance

**Figure 3:**
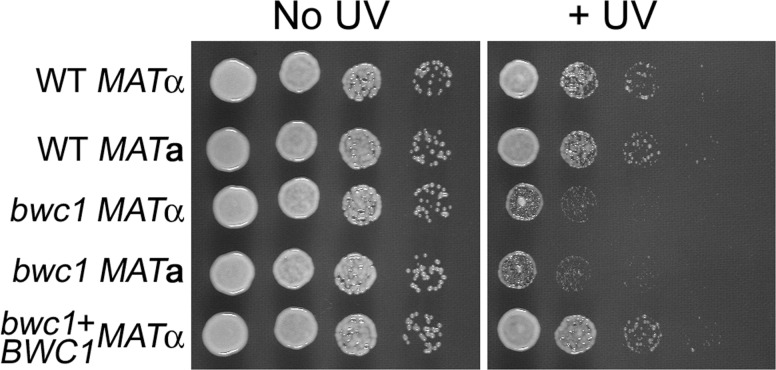
*bwc1* Mutants Are Hypersensitive to UV Light Ten-fold serial dilutions of log-phase yeast cells of *bwc1* mutant or wild type (WT) were plated in duplicate on YPD medium, and one plate was UV irradiated (~48 mJ/cm^2^). Reintroduction of a wild-type copy of the *BWC1* gene into the *bwc1* + *BWC1* mutant strain restores UV sensitivity to the wild-type level.

To search further for a function of *OPS1, PHY1,* and *BWC1,* growth of the single- and multiple-mutant strains was examined under a variety of in vitro conditions. The mutations had no effect on previously identified attributes required for virulence in mammalian hosts, such as the production of the pigment melanin or the polysaccharide capsule, or growth at 37 °C (unpublished data). The *ops1* and *phy1* mutant strains were as sensitive to UV light as wild type, but the *bwc1* mutants were markedly hypersensitive to UV light (Figure 3). Reintroduction of a wild-type copy of the *BWC1* gene into the *bwc1* mutant strain restored a wild-type level of sensitivity to UV light. Based on studies in other organisms, one target gene could be that encoding photolyase. However, there is no evidence for a photolyase in *C. neoformans,* based on the lack of photoreactivation and the absence of a homolog in genome databases (unpublished data). We conclude that Bwc1 functions in response to blue light to inhibit mating, and to UV light to regulate resistance to UV irradiation.

### *BWC1* Function Is Conserved between Two Varieties of C. neoformans

C. neoformans is a species complex of three divergent varieties or sibling species. C. neoformans var. *neoformans* (serotype D), utilized in the experiments described thus far, is commonly studied because of the ready availability of established congenic mating partners. However, this variety is uncommon in clinical settings (representing only 5% of cases), and we therefore re-isolated the *bwc1, ops1,* and *phy1* mutations in the most common pathogenic type, C. neoformans var. *grubii* (serotype A), in which congenic strains have only recently been developed [26]. Mating of serotype A laboratory strains is less efficient than that of the congenic serotype D strains, and their growth in the light is limited on the V8 (pH 5) medium used for genetic crosses. Serotype A *bwc1* bilateral crosses mated better in the dark than wild-type strains. Crosses performed in the light rarely resulted in filaments and were observed to do so only in the *bwc1* × *bwc1* mutant bilateral crosses (unpublished data). Using V8 (pH 7) medium and the serotype D *bwc1* strains as mating type tester strains, the effects of light on mating efficiency of serotype A could be more readily established. When crossed to serotype D *bwc1* strains, wild-type serotype A strains yielded fewer filaments than the *bwc1* mutant strains in both the light and dark, demonstrating a role for suppression of filament formation by *BWC1* under both conditions (see Figure 1D). The serotype A *bwc1* strains were also found to be hypersensitive to UV light (unpublished data). Reintroduction of the serotype A *BWC1* gene complemented the mutant phenotypes of both the serotype A and D *bwc1* mutants (Figures 1 and 3). In summary, *BWC1* function is conserved in two divergent cryptococcal varieties, and data derived from experiments on laboratory strains are also of significance to clinical isolates.

### Insertional Mutagenesis of a Novel Self-Filamentous Haploid Strain Identifies Other Components Required for Light Responses

**Figure 4:**
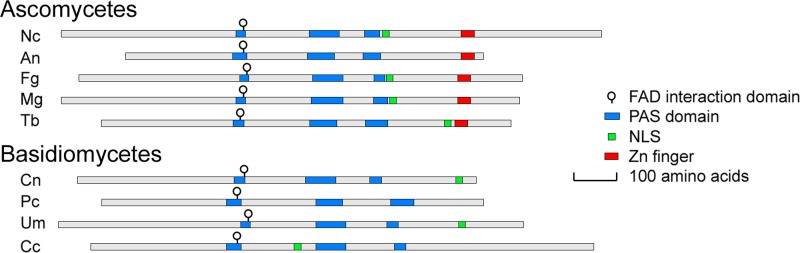
Ascomycete White Collar 1 Homologs Contain a Zinc Finger Domain; The Basidiomycete Homologs Do Not Comparison of the structure of the predicted WC-1 proteins from the ascomycetes N. crassa (Nc), Aspergillus nidulans (An), Magnaporthe grisea (Mg), Fusarium graminarium (Fg), and Tubor borchii (Tb), and the basidiomycetes C. neoformans (Cn), Coprinus cinereus (Cc), Ustilago maydis (Um), and Phanerochaete chrysosporium (Pc). Other domains are PAS (*PER*, *ARNT*, *SIM*) and NLS (nuclear localization signal), and the specialized PAS domain that interacts with the chromophore FAD is marked. Sequences are from GenBank (Nc, X94300; An, AF515628; Tb, AJ575416), the Broad Institute (Mg, Cn, Fg, Cc, Um), or the Department of Energy (Pc).

In contrast to the N. crassa WC-1 protein, the predicted C. neoformans Bwc1 protein has no zinc finger DNA binding domain or any other known DNA binding motif. Matches to *wc-1* were obtained from other fungi and the predicted proteins examined for these domains (Figure 4). The proteins share a similar structure with regard to the PAS domains, and all of the ascomycete *wc-1* genes examined contained a zinc finger DNA binding domain, whereas none of the basidiomycete *wc-1* homologs encode products with this domain. This suggests that the structural differences between the homologs are conserved in each phylum and are not unique to C. neoformans.

**Figure 5:**
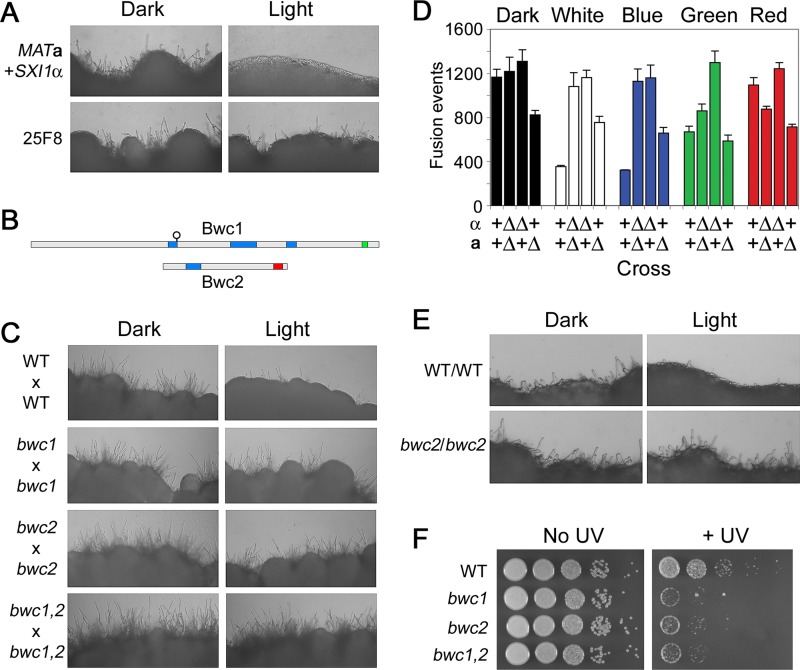
The *BWC2* Gene also Mediates UV/Blue Light Responses in C. neoformans (A) A self-filamentous haploid strain (MAT**a** +*SXI1α*) exhibits light-repressed filamentation. This strain was mutated by *Agrobacterium*-mediated T-DNA insertion, and insertional mutant strain 25F8 filaments equally well in the light and the dark. (B) Comparison of the structures of Bwc1 and Bwc2. The Bwc1 predicted protein (1,097 amino acids) has a LOV domain, two additional PAS domains and a nuclear localization signal. The Bwc2 predicted protein (392 amino acids) has a PAS domain and zinc finger DNA binding domain. (C) Bilateral mating between wild-type (WT), *bwcl, bwc2,* or *bwc1 bwc2* double *(bwc1,2)* mutant strains on V8 medium in the dark and the light (48 h). Filamentation is repressed in wild-type crosses in the light, but not in crosses between mutants or in the dark. (D) Fusion efficiency of strains under different wavelengths of light. Matings were between wild-type (+) partners, *bwc2* mutant (Δ) partners, or one wild-type and one mutant partner (*bwc2* α × WT **a**, or WT α × *bwc2* **a**). Mutation of *bwc2* in either or both mating partners relieves inhibition of fusion by white or blue light. Bars indicate the standard error of the mean of three replicates. (E) Filament formation in wild-type or *bwc2*/*bwc2* mutant diploid strains (24 h). Light does not repress filament formation in *bwc2*/*bwc2* diploids. (F) The *bwc2* and double *bwc1 bwc2* (*bwc1,2*) mutants are as hypersensitive to UV light as *bwc1* mutants. Ten-fold serial dilutions of yeast cells were plated in duplicate onto YPD medium, and one set was UV irradiated (~48 mJ/cm^2^).

We hypothesized that Bwc1 binds to an interacting DNA binding protein, because Bwc1 would be unable to act as a transcription factor on its own. Systematic deletion of all of the C. neoformans transcription factors, assuming these were annotated, is not technically feasible at this stage. Similarly, standard insertional mutagenesis poses a problem because the filamentation phenotype requires that both the MAT**a** and MATα mating partners bear the same mutation. However, overexpression of a mating type-specific homeodomain protein in a haploid strain of the opposite mating type confers a self-filamentous morphology [27]. We reasoned that such a self-filametous strain could be employed to perform random insertional mutagenesis, and devised a screen to identify the hypothetical protein interacting with Bwc1. The *SXI1α* gene, which encodes the MATα-specific homeodomain protein [27], was introduced into the genome of a MAT**a** haploid strain. The resulting MAT**a** +*SXI1α* strain (AI49) exhibited self-filamentous growth that was regulated by temperature, light, and nutrients. The strain grew as a budding yeast at 37 °C and filamented at 25 °C, and, like MAT**a**/MATα diploids, filamentation was inhibited by light, and was most robust on V8 mating medium (Figure 5A).

The self-filamentous MAT**a** +*SXI1*α haploid strain was mutated with transfer DNA (T-DNA) containing a nourseothricin resistance cassette using the trans-kingdom DNA delivery vehicle Agrobacterium tumefaciens. Then 2,715 individual mutant strains were isolated into 96-well microtiter plates and were analyzed with a stereomicroscope to examine filament formation after 24 and 48 h of growth in the dark and light on V8 agar medium. Three strains were isolated from this mutant library with a phenotype analogous to the *bwc1* mutant in that they filamented equally well in the light and dark and were UV-hypersensitive. The DNA regions flanking the T-DNA insertion were obtained by inverse PCR and compared to the C. neoformans genome database. One insertion (strain 1B4) is in a predicted gene with no database similarities (GenBank EAL21986). This gene was also identified in an independent insertion mutant with a different phenotype, and was therefore not analyzed further. The second isolate (28H3) bears an insertion in the promoter of the *RUM1**a*** gene, which is located in the mating type locus of C. neoformans [28]. Intriguingly, the *Ustilago maydis RUM1* homolog regulates transcription of the bE and bW homeodomain proteins, as well as of genes regulated by the bE/bW homeodomain complex [29]. The third isolate (25F8) contains an insertion in the promoter of a gene, designated *BWC2* (for a consistent nomenclature with respect to *BWC1;* GenBank AY882438), which encodes a predicted protein with a PAS and a zinc finger DNA binding domain (Figure 5B). Importantly, the predicted structure of the Bwc2 protein is strikingly similar to that of the N. crassa WC-2 protein, which does not perceive photons directly but instead interacts physically with the light sensor WC-1 and acts as a transcription factor. We chose to examine this gene further because its structure suggested that it might physically and functionally interact with the C. neoformans Bwc1 protein.

### Disruption of *BWC2* Results in the Same Phenotype as *bwc1* Mutation

A *bwc2* mutant allele was isolated in a wild-type background by replacing the coding region with the nourseothricin resistance gene in a serotype D strain. MAT**a** *bwc2* single- and *bwc1 bwc2* double-mutant strains were isolated following genetic crosses. In bilateral crosses, the *bwc2* mutants exhibit enhanced mating in the light, whereas wild-type mating is repressed (Figure 5C). As in the case of *bwc1* mutants, the inhibition of cell fusion, and also of filament formation after fusion, were no longer repressed by light in *bwc2* mutants (Figure 5D and 5E). In addition, the *bwc2* mutants were also hypersensitive to UV light (Figure 5F). The blind and UV-hypersensitive phenotypes of the *bwc1 bwc2* double mutants are comparable to those of the *bwc1* and *bwc2* single-mutant strains. When a wild-type copy of the *BWC2* gene was reintroduced into the *bwc2* mutant strain, UV sensitivity and inhibition of mating by light were restored to the wild-type level (unpublished data). A serotype A mutant of *bwc2* was also isolated, and exhibited phenotypes similar to the serotype A *bwc1* mutant: enhanced mating with the serotype D *bwc2* tester strain in the light and UV sensitivity (unpublished data). Thus, the *bwc1, bwc2,* and *bwc1 bwc2* mutant strains all exhibit similar phenotypes, and the double-mutant phenotype is no more severe than that of the single mutants, supporting the hypothesis that the products of the two genes function in a common pathway.

### Bwc1 and Bwc2 Interact in the Yeast Two-Hybrid System

**Figure 6:**
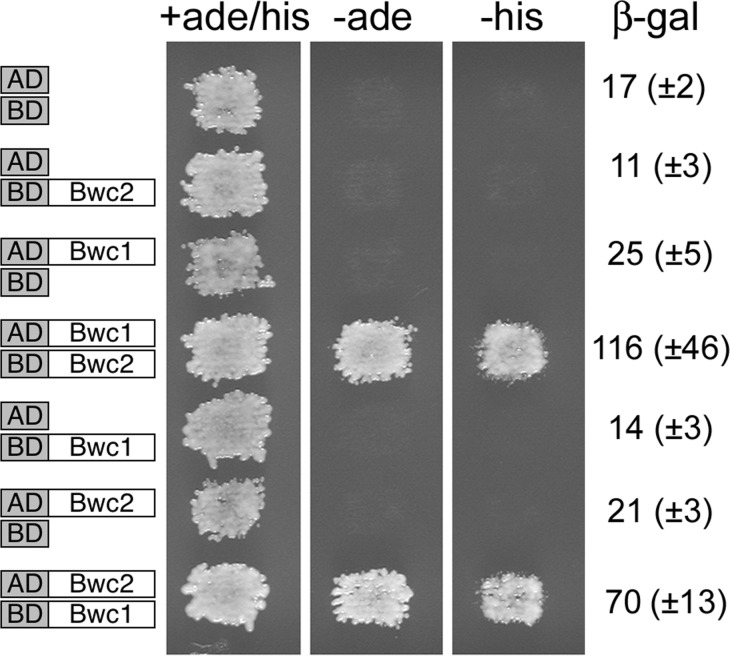
Bwc1 and Bwc2 Physically Interact The coding regions of the *BWC1* and *BWC2* genes were fused adjacent to the AD or BD of *S. cerevisiae GAL4*. Plasmids were cotransformed into a S. cerevisiae strain in which Gal4 regulates the *ADE2, HIS3,* and *lacZ* genes. Growth of strains in the absence of adenine (−ade) or histidine (−his) and increased β-galactosidase activity (β-gal, ± one standard error, Miller units) indicate protein-protein interactions.

A yeast two-hybrid analysis was conducted to test whether Bwc1 and Bwc2 physically interact. cDNA clones were fused to the S. cerevisiae Gal4 transcription factor activation (AD) or DNA binding (BD) domains and expressed in a S. cerevisiae strain in which the *GAL* promoter regulates *ADE2, HIS3,* and *lacZ* reporter genes. In S. cerevisiae strains expressing AD-Bwc1 and BD-Bwc2, or AD-Bwc2 and BD-Bwc1, the *ADE2, HIS3,* and *lacZ* reporter genes were all induced and the cells grew in the absence of adenine or histidine and expressed increased levels of β-galactosidase activity (Figure 6). In contrast, S. cerevisiae strains containing single Gal4-Bwc1/2 fusions and the corresponding Gal4 domain did not. These observations indicate that Bwc1 and Bwc2 can interact with one another in vivo. There was no evidence for homodimer formation for either Bwc1 or Bwc2, and no effects of light on the reporter gene-dependent growth of the strains were observed. Attempts to demonstrate Bwc1-Bwc2 interaction in C. neoformans itself via coimmunoprecipitation of epitope-tagged forms of Bwc1 and Bwc2 have been unsuccessful so far, due to the low abundance of the proteins, cross-reactivity of the antibodies with endogenous fungal proteins, and loss-of-function of tagged proteins in strains overexpressing these proteins (unpublished data). These findings demonstrate that Bwc1 and Bwc2 can physically interact when expressed in the nucleus of another fungal species, and that they can do so in a light-independent manner.

### Transcript Levels of *BWC2* Are Regulated by *BWC1* and Light

**Figure 7:**
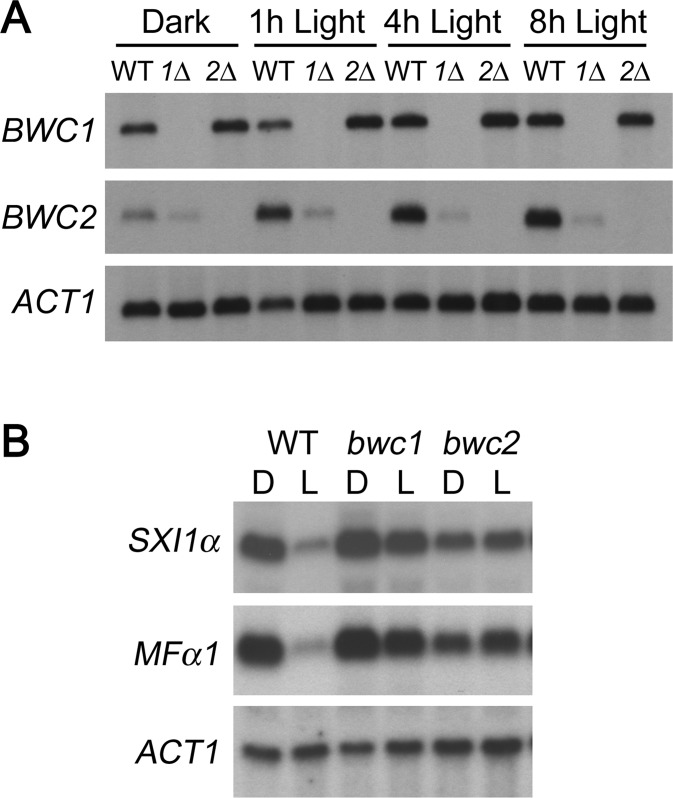
Transcript Analysis of *BWC1* and *BWC2,* and Their Effects on Genes Required for Mating (A) Transcript levels of *BWC2* are regulated by light, dependent on *BWC1*. Wild-type, *bwc1 (1*Δ*)* or *bwc2 (2*Δ*)* strains were inoculated onto V8 agar medium and wrapped in foil. A set of plates was removed from darkness 1 h, 4 h, and 8 h before the end of a 24-h period. Messenger RNA purified from 200 μg of total RNA isolated from these cultures was separated on an agarose gel, transferred to nitrocellulose, and probed with the *BWC1*, *BWC2,* and actin *(ACT1)* genes. No transcripts of *BWC1* or *BWC2* are observed in the *bwc1* or *bwc2* strains, respectively, consistent with the deletion strategy to create these strains. The transcript levels of *BWC1* are constant under these conditions. In contrast, *BWC2* transcript levels increase in the light, but not in strains bearing the *bwc1* mutation. (B) Bwc1 and Bwc2 regulate transcript levels of pheromone *MFα1* and homeodomain *SXI1α* genes. Crosses between wild-type (WT), *bwc1,* or *bwc2* mutant strains were conducted on V8 pH 7 medium, incubated in the light (L) or dark (D), and cells were harvested 24 h later. RNA was size-fractionated in agarose gels and blotted to nitrocellulose, and probed to detect pheromone *(MFα1)* or homeodomain *(SXI1α)* transcription, as well as actin *(ACT1)* as a control for RNA loading and transfer.

In *N. crassa,* light regulates transcript levels of *wc-1* but not *wc-2*. Transcription of *BWC1* and *BWC2* was assayed in the light and dark on V8 solid medium (Figure 7A). The levels of transcript, particularly of *BWC1,* were very low, and therefore samples were enriched approximately 20-fold by purifying mRNA from total RNA for Northern blot analysis. *BWC1* transcript levels were constant under these conditions. In contrast, *BWC2* is up-regulated in the presence of light, except in the *bwc1* mutant, demonstrating that *BWC2* is a light-regulated gene and dependent on the presence of *BWC1*. Thus, interestingly, the pattern of light induction of transcript levels is reversed between the two genes in C. neoformans compared to N. crassa.

### Transcript Levels of Genes Required for Mating Are Regulated by *BWC1* and *BWC2*

The mating phenotype of *bwc1* mutants suggested that Bwc1-Bwc2 regulates gene expression during mating. Transcript abundance of the pheromone gene *MFα1* and the homeodomain gene *SXI1α,* both of which are required for efficient mating and are known to be induced during mating and following cell fusion [21,27], was examined by Northern blot analysis of mating cultures grown in the light and dark for 24 h (Figure 7B). In crosses with *bwc1* and *bwc2* mutants, transcript levels were consistently high in both the light and the dark. In contrast, in crosses with wild-type parents the transcript levels of *MFα1* and *SXI1α* were reduced in the light compared to the dark (Figure 7B). These data suggest that Bwc1-Bwc2 function, directly or indirectly, to repress transcription of these two key genes that regulate mating and completion of the sexual cycle.

### *BWC1* and *BWC2* Regulate Virulence in Mammals

**Figure 8:**
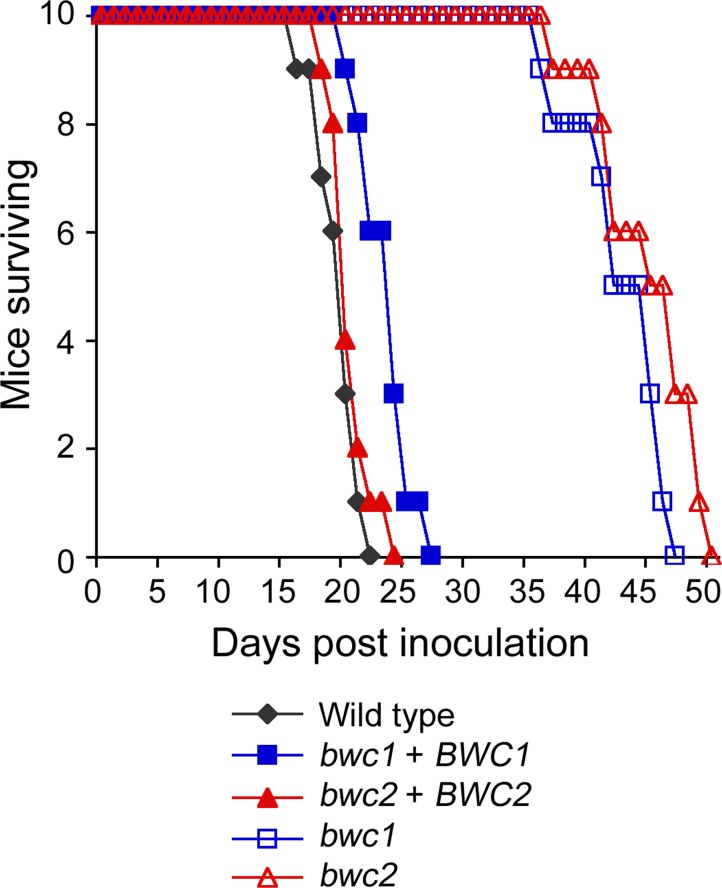
*BWC1* and *BWC2* Are Required for Full Virulence of C. neoformans in a Mammalian Host Ten mice each were infected intranasally with 1 × 10^5^ cells of wild-type, *bwc1* mutant, and *bwc2* mutant, and reconstituted (*bwc1* + *BWC1*; *bwc2* + *BWC2*) serotype A strains, and survival monitored daily. Mice infected with the wild-type and complementing strains progress to severe morbidity at the same rate, whereas mice infected with the *bwc1* or *bwc2* mutant strains survived twice as long.

C. neoformans is a pathogenic fungus that causes disease in humans and other animals. The wild-type, *bwc1,* and *bwc2* mutants, as well as the *bwc1* + *BWC1* and *bwc2* + *BWC2* complemented strains, were inoculated by inhalation into ten mice each, and host fitness and survival were examined daily (Figure 8). Animals infected with the wild-type or the *bwc1* + *BWC1* or *bwc2* + *BWC2* strains all died within 30 d of inoculation (average survival = 20.5 d, 24.4 d, and 21.5 d, respectively). In contrast, the mice infected with the *bwc1* or *bwc2* mutant strains were all healthy at 30 d after inoculation, and the first animal in these two groups that became moribund did so at day 37 (average survival = 43.2 and 45.1 d for *bwc1* and *bwc2* mutants, respectively). Bwc1 and Bwc2 are therefore not essential for virulence, but do contribute to the rate with which the fungus causes disease in the mammalian host. Thus, in addition to regulating development, Bwc1-Bwc2 also promote virulence.

## Discussion

Light inhibits both mating and a related differentiation process known as haploid fruiting in C. neoformans. Two approaches were employed to identify genes regulating these responses to light. First, we examined the genome of C. neoformans to identify homologs of genes involved in light perception in other organisms. Second, we designed a novel strategy to identify genes regulating sexual differentiation, and used a self-filamentous haploid strain in an insertional mutagenesis screen to define novel genes with roles in light responses.

*Opsin, phytochrome,* and white collar 1 homologs were found in the C. neoformans genome, and the function of these candidate photoreceptors was examined by gene disruption. No phenotypes were conferred by the *ops1* or *phy1* mutations, but deletion of the *wc-1* homolog *BWC1* abolished the inhibition of mating and haploid fruiting by light. The *bwc1* mutant phenotypes in the clinical background were generally equivalent to those observed in serotype D; however, in the serotype A crosses with the *bwc1* mutants, it was clear that mating inhibition occurred with a wild-type copy of *BWC1* regardless of the light status. In serotype D, inhibition of mating by light was shown to occur at both the cell fusion and the hyphal developmental stages. Cell fusion assays revealed that only one parent requires a *bwc1* mutation to circumvent repression by light, and the release from light repression in fusion is equivalent between unilateral (*bwc1* × wild type) and bilateral (*bwc1* × *bwc1*) crosses. This observation suggests that during mating only one cell, independent of mating type, needs to commit to fusion. Because a wild-type level of fusion was observed in unilateral crosses, rather than an expected 50% reduction, there must also be cross-talk between the two cells prior to fusion, which is probably mediated via pheromone sensing. Analysis of diploid strains showed that once cell fusion has occurred, the wild-type Bwc1 allele of the protein has sufficient activity, even in the heterozygous state, to repress filament formation in the presence of light to a level equivalent to that observed in wild-type diploid strains.

In an assay for the wavelength responsible for inhibition of cell fusion, blue light (rather than green or red wavelengths) was found to reduce fusion efficiency between strains with an intact copy of *BWC1*. No inhibition by white or blue light was observed during fusion of *bwc1* mutant strains. These data lead us to hypothesize that Bwc1 functions as a blue light photoreceptor, as is the case for N. crassa WC-1 [11,12]. To test this hypothesis, we initiated efforts to analyze the photochemistry of Bwc1. However, recombinant Bwc1 or fragments of Bwc1 expressed in E. coli cells were either produced in low quantities or were largely insoluble (unpublished data), and thus formal demonstration of photoreceptor function for Bwc1 remains to be established.

The *bwc1* mutants were also hypersensitive to UV light, showing that the Bwc1 protein functions in response to both blue (approximately 400–500 nm) and UV light (approximately 200–400 nm) wavelengths. The ability of blue or UV light to induce carotenoid formation in N. crassa was first noted a century ago [30]. Subsequent work has focused on light in the blue wavelengths, which is sensible given that any study on UV light and its regulation of fungal development is likely to be complicated by the effects this radiation has on cell viability and media stability. Nevertheless, there is evidence that N. crassa also perceives UV light through WC-1. Prior to cloning the *wc-1* gene, the spectra for inhibition of circadian rhythm and for photoinduction of carotenoid production were found to lie in both the UV and blue wavelengths [31,32]. Light treatment of N. crassa changes the light absorbance properties of mycelia, and the action spectrum of this response is within both the UV and blue wavelengths and closely overlaps that of flavins, with respective peaks at 360 and 470 nm [33]. In particular, the action spectra from physiological data overlap with the properties of the WC-1 protein purified from N. crassa cells, as WC-1-FAD or the chromophore FAD alone show two equal excitation peaks, one at 370 nm (UV) and one at 450 nm (blue) [11,12]. These data suggest that N. crassa WC-1 may also be a UV-responsive protein and function like C. neoformans in protecting the fungus from UV damage. The induction of UV-protecting carotenoids in N. crassa by light in a WC-1-dependent manner supports this hypothesis. Nevertheless, a UV-sensing function for the White collar proteins remains to be demonstrated through analysis of protein photochemistry and spectral and phenotypic analysis.

The predicted Bwc1 protein lacks a DNA binding domain found in the ascomycete WC-1 homologs. We hypothesized that there must be a second protein that interacts with Bwc1, and set out to identify this component via random insertional mutagenesis. To create a haploid self-filamentous strain of C. neoformans, we expressed the MAT-specific Sxi1α homeodomain protein in a MAT**a** haploid cell, resulting in robust induction of filament development. The self-filamentous strain was mutated with T-DNA insertions from *Agrobacterium,* and three mutant C. neoformans strains with equivalent filament formation in the light and the dark, and UV hypersensitivity, were isolated. In one strain, the T-DNA insertion lies in the promoter of a gene we designated *BWC2*, which has an analogous structure to the *N. crassa wc-2* gene (a PAS domain and zinc finger DNA binding domain) but shares much less sequence similarity relative to that between *C. neoformans BWC1* and *N. crassa wc-1*. The *BWC2* gene was not found in the initial candidate gene search because of this low sequence similarity and because the intron structure of C. neoformans confounded its identity. The *BWC2* gene was mutated to analyze its function. The *bwc2* and *bwc1 bwc2* double mutants exhibit phenotypes comparable to *bwc1* single mutants, and were nonresponsive to light during mating and haploid fruiting and hypersensitive to UV irradiation, suggesting they function in the same pathway. Furthermore, Bwc1 and Bwc2 interact in the yeast two-hybrid system, supporting a model in which the two proteins represent the integral components of a regulatory complex controlling light-regulated development.

The mating type loci of basidiomycetes have been well studied, and comprise two distinct gene sets: those that encode pheromones and those that encode homeodomain proteins, both of which control different steps in mating [34,35]. We hypothesized that transcription of the C. neoformans pheromone or homeodomain genes would be controlled via Bwc1-Bwc2. We focused on the pheromone genes, because they are important cell-cell signaling molecules, and because *mfα1,2,3* triple-mutant strains exhibit a reduction in fusion efficiency similar to that seen in *bwc1* mutant strains [21]. In N. crassa, transcription of the pheromone genes is under control of the circadian clock and presumably *wc-1* [36]. The mating type specific homeodomain protein Sxi1α of C. neoformans is important for events after cell fusion [27]. Examination of the transcription of *MFα1* and *SXI1α* in the light and dark in wild type compared to *bwc1* or *bwc2* crosses at 24 h showed that the *MFα1* and *SXI1α* genes are repressed by Bwc1-Bwc2 in the light. It is therefore likely that Bwc1-Bwc2 control mating by influencing the temporal regulation of these genes.

The roles of the *C. neoformans BWC1* and *BWC2* genes in virulence were examined. We hypothesized that the fungus may be able to sense darkness within the mammalian host and use this as a signal (possibly via Bwc1-Bwc2) to induce virulence. We also tested virulence, because several signal transduction pathways affecting C. neoformans mating also have an impact on virulence. Disruption of both *BWC1* and *BWC2* reduced the ability of C. neoformans to cause disease, as mice infected with the *bwc1* or *bwc2* mutant strains survived twice as long as those infected with wild-type or control strains. The Bwc1-Bwc2 system represents a novel class of protein complex that is required for cellular responses to an environmental stimulus and affects both development (mating) and virulence in pathogenic fungi [20]. In contrast to the cAMP signaling and calcineurin pathways, where mutants have reduced mating efficiency and virulence, here the *bwc1* and *bwc2* mutants have enhanced mating yet reduced virulence. In *bwc1* or *bwc2* strains there was no reduction in those traits normally associated with C. neoformans virulence, such as production of melanin or capsule, or growth at 37 °C or on minimal media. Identification of the downstream targets for this complex should further elucidate the molecular basis for its role in both mating and virulence.

**Figure 9:**
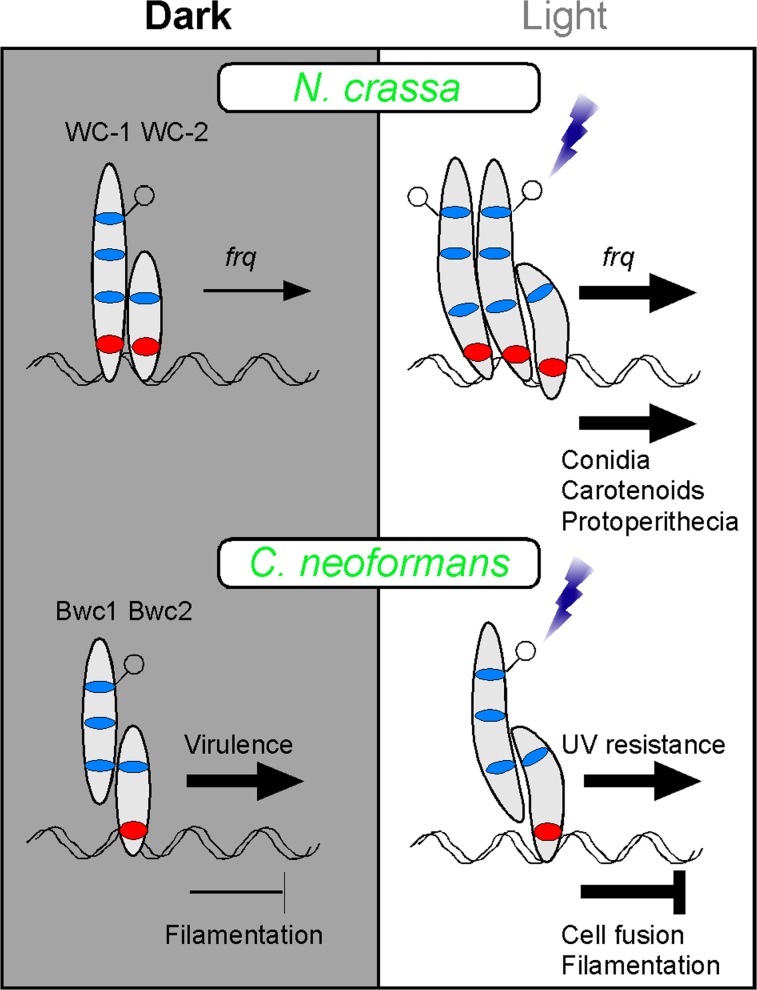
A Model of How Two Fungi May Respond to Light The Bwc1-Bwc2 interaction of C. neoformans shares conserved features with the WC-1-WC-2 interaction of N. crassa but also exhibits unique functional characteristics. In this model, C. neoformans Bwc1-Bwc2 bind to DNA in the dark and act as weak repressors to reduce filament development. We hypothesize that photons perceived through a proposed flavin moiety on Bwc1 cause a conformational change that increases repression of filament formation and cell fusion, and activates transcription of genes required for UV resistance. Alternatively, UV sensitivity may be mediated through repression by Bwc1-Bwc2 of a repressor protein. The N. crassa model is simplified from [3]: a complex of two units of WC-1 forms in response to light to cause an initial up-regulation of *frq* transcription above the levels occurring in the dark (FRQ feedback inhibits the White collar complex). The complex also increases transcription of genes required for other processes.

We propose a model for Bwc1-Bwc2 function that is similar to that of WC-1-WC-2 of N. crassa but differs in several key features (Figure 9). In this model, C. neoformans Bwc1-Bwc2 bind to DNA in the dark and act as weak repressors to reduce filament development. We hypothesize that photons perceived through a flavin cofactor cause a conformational change that enhances repression of filament formation and cell fusion, and activates transcription of genes required for UV resistance/DNA repair. It is also formally possible that light causes Bwc1-Bwc2 to increase transcription of a gene that functions to repress mating, and/or represses transcription of a repressor of UV sensitivity. The N. crassa model is similar but differs in several key features. Recent evidence suggests that a complex of two subunits of WC-1 and one subunit of WC-2 form in response to light [3,10]. The complex positively regulates transcription of genes required for conidiation, mating, and carotenoid production, in marked contrast to the negative regulation of mating observed in C. neoformans. Another difference is that *wc-1* is light-regulated in N. crassa, while *BWC2* is light-regulated in C. neoformans and *BWC1* is not. The N. crassa complex also regulates transcription of *frq,* and the FRQ protein feedback inhibits the complex, thereby contributing to the wiring of the circadian clock. The roles for WC-1 and WC-2 in N. crassa photoperception also change during the day, adding to the challenge of elucidating their functions. Future studies in C. neoformans will define downstream targets of Bwc1-Bwc2, regulation of Bwc1 and Bwc2 and their complex, and the creation of alleles of Bwc1 bearing mutations in the predicted flavin interacting domain to elucidate the proposed roles of these proteins in light perception.

Components of the White collar sensing system have been identified in other fungi (see Figure 5), and are likely to function in light responses in these and other fungal species. Recently the Trichoderma atroviride homologs of N. crassa WC-1 and WC-2 were isolated and mutated, demonstrating a role for these genes in light-induced conidiation and the induction of photolyase [37]. A gene homologous to *BWC1* was identified in the model basidiomycete C. cinereus as mutated in a strain defective in light-regulated development of the mushroom cap [38]. Developmental regulation in C. cinereus is blue-light dependent [39,40], and UV/blue light also regulate development of numerous other fungi (for review see [41]). A homolog of *wc-1* was identified in the truffle-forming ascomycete *Tubor bruchii,* in which blue light inhibits hyphal growth [42]. Thus, this gene and its homologs may have applications even to cultivated, edible fungi. It will be of interest to establish whether White collar-like proteins are present in the genomes of the two other fungal phyla proposed for genome sequencing, the zygomycetes and the chytrids. The responses of the zygomycete *Phycomyces* to light, particularly blue and UV wavelengths, have been well characterized, and numerous mutants (e.g., ten different *mad* mutants) affecting responses or sensitivity to particular wavelengths have been isolated, but no photoreceptor genes have as yet been identified [2]. We predict that some of these known mutations will be found to affect White collar homologs.

Finally, we speculate that White collar genes could be of major significance for terrestrial life. The discovery of the *BWC1* and *BWC2* genes as potential UV-blue light responsive proteins in a basidiomycete indicates that this type of protein complex is ancient in the fungal kingdom. The fossil record shows a clear divergence of the fungal kingdom into the four phyla by the Devonian [416–359 million years ago (mya)], and a Precambrian origin (prior to 542 mya) for the fungi has been suggested [43,44,45,46]. Margulis et al. proposed that sexual recombination and DNA repair were coselected in the Precambrian for protection against UV light [47], and genes are known that control both recombination and sensitivity to UV light. Bwc1-Bwc2 in C. neoformans regulates both UV sensitivity and sexual development, ultimately leading to recombination. During the Silurian division (416–444 mya), the UV-protection role of the WC-1 proteins could have conferred a major selective advantage to the fungi when they and plants cocolonized the continents at a time when there was no shade from solar radiation. The proteins could have been especially important at other times of global ecological change associated with elevated UV irradiation due to atmospheric and vegetation changes, such as at the end of the Permian (250 mya) or Cretaceous (65 mya), when there are spikes of fungi in the fossil record [48,49]. The UV-protecting ability of the WC-1 proteins is a likely selective force that has served to maintain their presence in fungi to this day.

## Materials and Methods

### 

#### Gene identification and fungal strains

Candidate photoreceptors were identified in the C. neoformans genome projects. Gene transcription was tested using RT-PCR and rapid amplification of cDNA ends (RACE) with the GeneRacer Kit (Invitrogen, Carlsbad, California, United States). Three genes were mutated in serotype D strain JEC43 (MATα *ura5*) and serotype A strain JF99 (MAT**a** *ura5*). Mutations were made by biolistic introduction of disruption alleles generated by overlap PCR with 1.5-kb DNA on either side of the *URA5* gene [50,51]. *BWC2* was disrupted using a nourseothricin resistance cassette [52,53] to replace the gene in the serotype D strain JEC21 or the serotype A strain KN99α (both MATα). Gene disruption was confirmed by PCR and Southern blot analysis using standard methods. The mutant serotype D strains were crossed to the congenic strain JEC20 (MAT**a**) to obtain strains with the opposite mating type. Through a series of crosses, strains with double or triple mutations in both mating types were isolated. A set of strains with auxotrophic markers (either *lys1* or *ade2*) with the mutation or wild type at the *BWC1* locus was also generated by crossing. The serotype A *bwc1::URA5* strain was crossed to strain KN99α to obtain a MATα *bwc1* strain. For reconstitution, the *BWC1* or *BWC2* genes were amplified with primers JOHE8744 and JOHE8745 or JOHE12641 and JOHE12642, respectively, from genomic DNA of strain H99, and ligated adjacent to a cassette conferring resistance to neomycin (G418). A linearized version of this vector was introduced into *bwc1* or *bwc2* mutant strains. The self-filamentous serotype D strain was obtained by introducing the *SXI1α* gene adjacent to *URA5* into strain JEC34 (MAT**a** *ura5*). This strain was mutated with *Agrobacterium*-mediated integration of T-DNA containing the *NAT* gene [52,54]. Strains created and primers used in strain construction are listed in Tables S1 and S2.

#### Phenotypic analysis of mutant strains

Strains were compared to each other and reference laboratory strains for the ability to mate in the presence of white fluorescent light (1,500–3,500 lux) or darkness on V8 medium at pH 5 (serotype A) or pH 7 (serotype A and D). The growth of strains was also examined at 37 °C on YPD medium, for melanin production on bird seed agar (70 g/L ground bird seed, 0.1% glucose, 0.05% Tween-20) or on low-glucose (0.1%) medium supplemented with the diphenolic molecule L-DOPA (100 mg/ml). Capsule production was assayed by growing strains in liquid medium with low levels of glucose (0.5%) and iron (20 mg/L of the chelator EDDHA), and examining exclusion of India ink particles from fungal cells. Strains were grown to logarithmic phase in liquid YPD medium and serial dilutions spotted onto YPD agar plates, which were then irradiated with UV light (0.2 min setting, approximately 48 mJ/cm^2^; UV Stratalinker 2400, Stratagene, La Jolla California, United States) to test for UV sensitivity.

#### Wavelength of inhibition and analysis of light inhibition during stages of mating

Crosses between *lys1* or *ade2* auxotrophic strains with or without the *bwc1* or *bwc2* mutations were conducted under illumination modified with filters to provide blue, green, or red light (LE 4747 blue, LE 4758 green and LE 4725 red; Calumet, Bensenville Illinois, United States). Yeast cells (1 × 10^7^/ml) were inoculated in 5-μl drops onto V8 (pH 7) medium, and 24 or 48 h later the mating mix scraped from the surface, and cells were resuspended in sterile water and plated onto minimal medium (yeast nitrogen base; YNB) to select for prototrophs that result from fusion events. Stable diploid yeast strains were created by incubating the cells at 37 °C on YNB medium.

#### Transcription analysis

Strains (1.25 × 10^8^ cells) were inoculated onto 15-cm diameter petri dishes containing V8 pH 7 medium, which induces mating. Cells were scraped from the surface 24 h later, frozen, and lyophilized. Total RNA was isolated from cells using TRIzol reagent (Invitrogen) according to manufacturer's instructions. Messenger RNA was isolated from 200 μg of total RNA using the PolyATtract isolation system (Promega, Madison, Wisconsin, United States). RNA was separated on denaturing agarose gels, blotted to nitrocellulose (Zeta-Probe, Bio-Rad, Hercules California, United States), and probed with [^32^P]-dCTP-radiolabeled DNA fragments. Probes comprising *ACT1* (encoding actin)*, BWC1, BWC2, SXI1α,* and *MFα1* genes were amplified from genomic DNA (primers in Table S2). Crosses comprised wild-type strains JEC20 x JEC21, *bwc1* mutant strains AI5 (MATα) × AI6 (MAT**a**)*,* or *bwc2* mutant strains AI76 (MATα) × AI78 (MAT**a**)*,* and were maintained in constant light or dark. For analysis of *BWC1* and *BWC2* transcription, cultures of wild-type, *bwc1,* or *bwc2* (strains JEC21, AI5, and AI76, respectively) were wrapped in aluminum foil and exposed to light 0 h, 1 h, 4 h, or 8 h prior to the end of a 24-h incubation.

#### Yeast two-hybrid system

cDNAs of *BWC1* and *BWC2* were amplified either by overlap PCR from genomic DNA or from RT-PCR from RNA, and sequenced to identify clones without errors. Products were cloned into plasmids pGBD.c1 and pGAD.c1, and the S. cerevisiae reporter strain PJ69–4A was cotransformed with plasmids using the lithium acetate/heat shock method [55]. Double transformants were selected on media lacking leucine and tryptophan. Interactions were assessed by growth in the absence of adenine or histidine (+ 5 mM 3-aminotriazole) and β-galactosidase assays [56].

#### *C. neoformans* virulence assay

For murine killing assays, serotype A C. neoformans cells were used to infect 25-g female A/Jcr mice (NCI/Charles River Laboratories, Frederick, Maryland, United States) by nasal inhalation [57]. Ten mice were inoculated each with a 50-μl drop containing 1 × 10^5^ yeast cells of KN99α, *bwc1, bwc1* + *BWC1* reconstituted, *bwc2* and *bwc2* + *BWC2* reconstituted strains. Survival data were analyzed with a logrank test to determine statistical significance. The murine experiment protocol was approved by the Duke University Animal Use Committee.

## Supporting Information

Table S1 Cryptococcus *neoformans* strains Mutant alleles were created by replacing the coding region of genes with markers to complement uracil auxotrophy *(URA5)* or confer resistance to nourseothricin *(NAT)*. Mutations were complemented by reintroduction of wild-type copies of the gene fused to a gene conferring resistance to neomycin *(NEO)*.
(58 KB DOC).

Table S2 Oligonucleotides Primers used to create gene disruption alleles, probes, and clones for yeast two-hybrid assays.
(50 KB DOC).

### Accession Numbers

The GenBank (http://www.ncbi.nlm.nih.gov/) accession numbers of the genes discussed in this paper are *Aspergillus nidulans wc-1* (AF515628), *BWC1* (AY882437), *BWC1* (AY882438), *N. crassa wc-1* (X94300), *OPS1* (AY882440), *PHY1* (AY882439), and *T. borchii Tbwc-1* (encodes wc-1 protein) (AJ575416). The Broad Institute (http://www.broad.mit.edu/annotation/fungi/fgi/) has sequence for White collar 1 homologs from *Coprinus cinereus, Fusarium graminearum*, Magnaporthe grisea, and Ustilago maydis. The Department of Energy (http://genome.jgi-psf.org/whiterot1/whiterot1.home.html) has the sequence for the White collar 1 homolog of Phanerochaete chrysosporium.

## Abbreviations

AD: activation domain
BD: binding domain
FAD: flavin adenine dinucleotide
LOV: light
mya: million years ago
PAS: Per-Arnt-Sim
T-DNA: transfer DNA
UV: ultraviolet
WC: White collar
