# Supplementary material for: Light Controls Growth and Development via a Conserved Pathway in the Fungal Kingdom
Source: PLoS Biol. 2005 Mar 15;3(4):e95. doi: 10.1371/journal.pbio.0030095 (PMC1064852; doi:10.1371/journal.pbio.0030095)
Supplement: Supplementary file 1 [file pbio.0030095.st001.doc]

**Supplementary Table I.** *Cryptococcus neoformans* strains created.

| Name | **Sero-type** | **Mating type** | **Genotype** | **Background/**  **parent** |
| --- | --- | --- | --- | --- |
| AI1 | D | α | *ops1::URA5 ura5* | JEC43 |
| AI2 | D | **a** | *ops1::URA5* | AI1 x JEC20 |
| AI3 | D | α | *phy1::URA5 ura5* | JEC43 |
| AI4 | D | **a** | *phy1::URA5* | AI3 x JEC20 |
| AI5 | D | α | *bwc1::URA5 ura5* | JEC43 |
| AI6 | D | **a** | *bwc1::URA5* | AI5 x JEC20 |
| AI7 | D | α | *ops1::URA5 phy1::URA5* | AI1 x AI4 |
| AI8 | D | **a** | *ops1::URA5 phy1::URA5* | AI1 x AI4 |
| AI9 | D | α | *phy1::URA5 bwc1::URA5* | AI4 x AI5 |
| AI10 | D | **a** | *phy1::URA5 bwc1::URA5* | AI4 x AI5 |
| AI11 | D | α | *ops1::URA5 bwc1::URA5* | AI1 x AI6 |
| AI12 | D | **a** | *ops1::URA5 bwc1::URA5* | AI1 x AI6 |
| AI13 | D | α | *ops1::URA5 phy1::URA5 bwc1::URA5* | AI7 x AI12 |
| AI14 | D | **a** | *ops1::URA5 phy1::URA5 bwc1::URA5* | AI7 x AI12 |
| AI18 | A | **a** | *ops1::URA5 ura5* | JF99 |
| AI20 | A | **a** | *phy1::URA5 ura5* | JF99 |
| AI21 | A | **a** | *bwc1::URA5 ura5* | JF99 |
| AI22 | A | α | *bwc1::URA5* | AI21 x KN99α |
| AI41 | D | α | *BWC1 ade2* | AI5 x JEC171 |
| AI42 | D | α | *bwc1::URA5 ade2* | AI5 x JEC171 |
| AI43 | D | **a** | *bwc1::URA5 lys1 NAT* | AI6 x JEC31-NAT |
| AI44 | D | **a** | *BWC1 lys1 NAT* | AI6 x JEC31-NAT |
| AI45 | D | **a**/α diploid | *BWC1*/*BWC1 lys1/LYS1 ade2/ADE2 nat/NAT* | AI41 x AI44 |
| AI46 | D | **a**/α diploid | *bwc1::URA5/bwc1::URA5 lys1/LYS1 ade2/ADE2 nat/NAT* | AI42 x AI43 |
| AI49 | D | **a** | *SXI1α-URA5 ura5* | JEC34 |
| AI51 | D | α | *bwc1::URA5* + *BWC1-NEO* | AI5 |
| AI52 | D | α | *bwc1::URA5* + *BWC1-NEO* | AI5 |
| AI50 | A | **a** | *bwc1::URA5* + *BWC1-NEO* | AI21 |
| AI59 | A | α | *phy1::URA5 URA5* | AI20 x KN99α |
| AI60 | A | **a** | *phy1::URA5 URA5* | AI20 x KN99α |
| AI76 | D | α | *bwc2::NAT* | JEC21 |
| AI78 | D | **a** | *bwc2::NAT* | AI76 x JEC20 |
| AI79 | D | α | *bwc1::URA5 bwc2::NAT* | AI5 x AI78 |
| AI80 | D | **a** | *bwc1::URA5 bwc2::NAT* | AI5 x AI78 |
| AI81 | A | α | *bwc1::NAT* | KN99α |
| AI82 | A | α | *bwc2::NAT* | KN99α |
| AI83 | D | α | *bwc2::NAT BWC2-NEO* | AI76 |
| AI86 | A | α | *bwc2::NAT BWC2-NEO* | AI82 |
| AI85 | A | α | *bwc1::NAT BWC1-NEO* | AI81 |
| AI87 | D | **a**/α diploid | *BWC1*/*bwc1::URA5 lys1/LYS1 ade2/ADE2 nat/NAT* | AI41 x AI43 |
| AI88 | D | **a**/α diploid | *bwc1::URA5/BWC1 lys1/LYS1 ade2/ADE2 nat/NAT* | AI42 x AI44 |

| AI91 | D | α | *BWC2 ade2* | AI76 x JEC171 |
| --- | --- | --- | --- | --- |
| AI92 | D | **a** | *bwc2::NAT lys2* | AI76 x JEC171 |
| AI93 | D | α | *bwc2::NAT ade2* | AI76 x JEC171 |
| AI94 | D | **a**/α diploid | LYS2/lys2 ADE2/ade2 | AI91 x JEC32 |
| AI95 | D | **a**/α diploid | LYS2/lys2 ADE2/ade2 bwc2::NAT/bwc2::NAT | AI92 x AI93 |
| AI96 | D | **a**/α diploid | LYS2/lys2 ADE2/ade2 BWC2/bwc2::NAT | AI91 x AI92 |
| AI97 | D | **a**/α diploid | LYS2/lys2 ADE2/ade2 BWC2/bwc2::NAT | AI93 x JEC32 |
| 1A3 | D | **a** | *eal21986::T-DNA-NAT SXI1α-URA5 ura5* | AI49 |
| 25F8 | D | **a** | *bwc2::T-DNA-NAT SXI1α-URA5 ura5* | AI49 |
| 28H3 | D | **a** | *rum1****a****::T-DNA-NAT SXI1α-URA5 ura5* | AI49 |

Mutant alleles were created by replacing the coding region of genes with markers to complement uracil auxotrophy (*URA5*) or confer resistance to nourseothricin (*NAT*). Mutations were complemented by reintroduction of wild type copies of the gene fused to a gene conferring resistance to neomycin (*NEO*).
