# Supplementary material for: Light Controls Growth and Development via a Conserved Pathway in the Fungal Kingdom
Source: PLoS Biol. 2005 Mar 15;3(4):e95. doi: 10.1371/journal.pbio.0030095 (PMC1064852; doi:10.1371/journal.pbio.0030095)
Supplement: Supplementary file 2 [file pbio.0030095.st002.doc]

**Supplementary Table II.** Oligonucleotides used.

| Primer name | Sequence (5’3’) |
| --- | --- |
|  |  |
| Disruption *OPS1* with *URA5* (serotype A and D) | |
| 1 JOHE8742 | AGCTTCGGCCTTGAGCATGAG |
| 2 JOHE8782 | GGTCGAGCAACTTCCTCCTGCGAAGTAATCCATCC |
| 3 JOHE8787 | GGATGGATTACTTCGCAGGAGGAAGTTGCTCGACC |
| 4 JOHE8788 | CAGAACCAACGCTGGTAACCTTGCCTCCAGGAGGTGG |
| 5 JOHE8783 | CCACCTCCTGGAGGCAAGGTTACCAGCGTTGGTTCTG |
| 6 JOHE8743 | TCGCCTATCAAACAGATTCTC |
|  |  |
| Disruption *PHY1* with *URA5* (serotype A and D) | |
| 1 JOHE8887 | TGGTCGTGGACAAGGTGATG |
| 2 JOHE8889 | GGTCGAGCAACTTCCTCCATTCTCTCCAATGCACG |
| 3 JOHE8888 | CGTGCATTGGAGAGAATGGAGGAAGTTGCTCGACC |
| 4 JOHE8891 | CGAAGGATTCAATTTCAGGCTTGCCTCCAGGAGGTGG |
| 5 JOHE8890 | CCACCTCCTGGAGGCAAGCCTGAAATTGAATCCTTCG |
| 6 JOHE8892 | GTGTAGGTGAGGAAGCAGC |
|  |  |
| Disruption *BWC1* with *URA5* (serotype A and D) | |
| 1 JOHE8744 | TAGAGCAAAGCGAAAAGCTGGG |
| 2 JOHE8771 | GGTCGAGCAACTTCCTCCATGCTATGACCGCTTAC |
| 3 JOHE8770 | GTAAGCGGTCATAGCATGGAGGAAGTTGCTCGACC |
| 4 JOHE8773 | GAGAGCCGCAATGCTATCCTTGCCTCCAGGAGGTGG |
| 5 JOHE8772 | CCACCTCCTGGAGGCAAGGATAGCATTGCGGCTCTC |
| 6 JOHE8745 | ACACGCCTACCTACGTGCTAGC |
|  |  |
| Disruption *BWC1* with *NAT* (serotype A) | |
| 1 JOHE8744 | TAGAGCAAAGCGAAAAGCTGGG |
| 2 JOHE12638 | GCTTATGTGAGTCCTCCCCATGCTATGACCGCTTAC |
| 3 JOHE8677 | GAAGAGATGTAGAAACGAG |
| 4 JOHE11866 | GGGAGGACTCACATAAGC |
| 5 JOHE12639 | CTCGTTTCTACATCTCTTGATAGCATTGCGGCTCTC |
| 6 JOHE8745 | ACACGCCTACCTACGTGCTAGC |
|  |  |
| Disruption *BWC2* with *NAT*-ST31 (serotype D) | |
| 1 JOHE11711 | GGAGGGAAAAGGAACCTTG |
| 2 JOHE11712 | GTCATAGCTGTTTCCTGCATTAGTTGACGATTCGC |
| 3 M13F | GTAAAACGACGGCCAG |
| 4 M13R | CAGGAAACAGCTATGAC |
| 5 JOHE11713 | CTGGCCGTCGTTTTACTGATAAGCAATCTTGAGAG |
| 6 JOHE11714 | CTAACTGGGCTGTTTCAATC |

| Disruption *BWC2* with *NAT*-ST31 (serotype A) | |
| --- | --- |
| 1 JOHE12641 | CTCCAGTTCTTCATCCAAGG |
| 2 JOHE11712 | GTCATAGCTGTTTCCTGCATTAGTTGACGATTCGC |
| 3 M13F | GTAAAACGACGGCCAG |
| 4 M13R | CAGGAAACAGCTATGAC |
| 5 JOHE12642 | CTGGCCGTCGTTTTACTACTCAGATGGGCAAAACG |
| 6 JOHE12643 | AAAGGAGGTAATGAAGGACC |
|  |  |
| Northern blot analysis | |
| *MF*α*1* (Hull et al., 2002) | |
| JOHE1204 | TTTTACGCTTTTTGCAGATTCCGCCAAA |
| JOHE3242 | GACCACTGTTTCTTTCGTTCT |
| *SXI1*α | |
| JOHE11262 | ATGCTTTCGACCTGTGATAG |
| JOHE11263 | GGTTTATCTGTATTAACACG |
| *BWC1* |  |
| JOHE9567 | TCAAGCCTCATCGTCAGC |
| JOHE9568 | GATGAAGTTCATACTGCCAC |
| *BWC2* |  |
| JOHE11715 | CATACATCAGGATGCTCTCTGC |
| JOHE11716 | AAGGACCCTTTCTCCATTCTGG |
| *Actin* | |
| JOHE8713 | ATGGAAGAAGAAGGTACG |
| JOHE8714 | TTAGAAACACTTTCGGTG |
|  |  |
| Creation of *SXI1*α-*URA5* construct | |
| 1 JOHE9876 | CGTAGGTGACTTCGGTATGG |
| 2 JOHE9877 | GGTCGAGCAACTTCCTCTGAATGATGAAACGTCGG |
| 3 JOHE9878 | CCGACGTTTCATCATTCAGAGGAAGTTGCTCGACC |
| 4 JOHE8773 | GAGAGCCGCAATGCTATCCTTGCCTCCAGGAGGTGG |
|  |  |
| *bwc1* cDNA construction for yeast two-hybrid assay | |
| 1 JOHE12180 | GGATCCATGAATTTCCAAACTCCTCC |
| 2 JOHE12187 | GTCATATCTCCTTATATCCACCACTACGAAACTAC |
| 3 JOHE12186 | GTAGTTTCGTAGTGGTGGATATAAGGAGATATGAC |
| 4 JOHE12189 | ATAAGGCATGTATGAAGTCATCCGTATTATCCAGAA |
| 5 JOHE12188 | TTCTGGATAATACGGATGACTTCATACATGCCTTAT |
| 6 JOHE12181 | GGATCCAATACCCCCAATTTGCGAGC |
|  |  |
| *bwc2* cDNA cloning for yeast two-hybrid assay | |
| JOHE12182 | GGATCCATGTCCCTCCTCGCCGAGTC |
| JOHE12183 | GGATCCAGATTGCTTATCTTTTTTGGG |

For disruption, combinations of primers 1 and 2, 5 and 6 were used on *C. neoformans* genomic DNA, and 3 and 4 on DNA of plasmids containing a selectable marker. Overlap PCR was conducted with primers 1 and 6 on the three products mixed together. BamHI sites are underlined in primers used for yeast two-hybrid plasmid construction.
